# A prospective 2-site parallel intervention trial of a research-based film to increase exercise amongst older hemodialysis patients

**DOI:** 10.1186/s12882-017-0454-4

**Published:** 2017-01-26

**Authors:** Pia Kontos, Shabbir M.H. Alibhai, Karen-Lee Miller, Dina Brooks, Romeo Colobong, Trisha Parsons, Sarbjit Vanita Jassal, Alison Thomas, Malcolm Binns, Gary Naglie

**Affiliations:** 1 Toronto Rehabilitation Institute-University Health Network, 550 University Ave., Toronto, ON M5G 2A2 Canada; 2 Dalla Lana School of Public Health, University of Toronto, 155 College St., Toronto, ON M5T 3M7 Canada; 3 Department of Medicine, University of Toronto, 1 King’s College Cir., Toronto, ON M5S 1A8 Canada; 4 Institute of Health Policy, Management and Evaluation, University of Toronto, 155 College St., Toronto, ON M5T 3M7 Canada; 5 Institute of Medical Sciences, University of Toronto, 1 King’s College Cir., Toronto, ON M5S 1A8 Canada; 6 Department of Medicine, University Health Network, 200 Elizabeth St., Toronto, ON M5G 2C4 Canada; 7 Department of Physical Therapy, University of Toronto, 500 University Ave., Toronto, ON M5G 1V7 Canada; 8 School of Rehabilitation Therapy, Queen’s University, 31 George St., Kingston, ON K7L 3N6 Canada; 9 Division of Nephrology, University Health Network, 200 Elizabeth St., Toronto, ON M5G 2C4 Canada; 10 St. Michael’s Hospital, 30 Bond St., Toronto, ON M5B 1W8 Canada; 11 Lawrence S. Bloomberg Faculty of Nursing, University of Toronto, 155 College St., Toronto, ON M5T 1P8 Canada; 12 Rotman Research Institute, Baycrest Health Sciences, 3560 Bathurst St., Toronto, ON M6A 2E1 Canada; 13 Department of Medicine, Baycrest Health Sciences, 3560 Bathurst St., Toronto, ON M6A 2E1 Canada

**Keywords:** End-stage renal disease (ESRD), Hemodialysis, Exercise, Knowledge translation, Education, Mixed methods

## Abstract

**Background:**

Evidence suggests that exercise training for hemodialysis patients positively improves morbidity and mortality outcomes, yet exercise programs remain rare and are not systematically incorporated into care. We developed a research-based film, *Fit for Dialysis,* designed to introduce, motivate, and sustain exercise for wellness amongst older hemodialysis patients, and exercise counseling and support by nephrologists, nurses, and family caregivers. The objective of this clinical trial is to determine whether and in what ways *Fit for Dialysis* improves outcomes and influences knowledge/attitudes regarding the importance of exercise for wellness in the context of end-stage renal disease.

**Methods/Design:**

This 2-site parallel intervention trial will recruit 60 older hemodialysis patients from two urban hospitals. The trial will compare the film + a 16-week exercise program in one hospital, with a 16-week exercise-only program in another hospital. Physical fitness and activity measures will be performed at baseline, 8 and 16 weeks, and 12 weeks after the end of the program. These include the 2-min Walk Test, Grip Strength, Duke Activity Status Index, and the Timed Up-and-Go Test, as well as wearing a pedometer for one week. Throughout the 16-week exercise program, and at 12 weeks after, we will record patients’ exercise using the Godin Leisure-time Exercise Questionnaire. Patients will also keep a diary of the exercise that they do at home on non-dialysis days. Qualitative interviews, conducted at baseline, 8, and 16 weeks, will explore the impact of *Fit for Dialysis* on the knowledge/attitudes of patients, family caregivers, and nephrology staff regarding exercise for wellness, and in what ways the film is effective in educating, motivating, or sustaining patient exercise during dialysis, at home, and in the community.

**Discussion:**

This research will determine for whom *Fit for Dialysis* is effective, why, and under what conditions. If *Fit for Dialysis* is proven beneficial to patients, nephrology staff and family caregivers, research-based film as a model to support exercise promotion and adherence could be used to support the National Kidney Foundation’s guideline recommendation (NKF-KDOQI) that exercise be incorporated into the care and treatment of dialysis patients.

**Trial registration:**

NCT02754271 (ClinicalTrials.gov), retroactively registered on April 21, 2016.

## Background

The prevalence of end-stage renal disease (ESRD) in Canada between 1991 and 2010 increased significantly with the largest growth being among older persons [1]. As a consequence of ESRD and its sequelae, patients often demonstrate reduced physical capacity and functional impairment [2–4]. These limitations are greater in older dialysis patients [5], the most rapidly growing segment of the dialysis population [6], and have been shown to compromise their rehabilitation potential [7].

Research demonstrates a number of potential benefits that hemodialysis (HD) patients may achieve from exercise. These include improvements in functional capacity [8–11], skeletal muscle efficiency [8, 9, 12], cardiac performance [13], blood pressure [14] and pulse pressure [15], and health-related quality of life [13], including a decrease in anxiety and depression [10]. Many of these outcomes are relevant for reducing patients’ risk for cardiovascular mortality as well as for improvements in physical functioning [8, 16]. Exercise has also been demonstrated to improve dialysis adequacy [15, 17] and increase long-term survival [18]. Although few studies specifically concern exercise in older HD patients, those conducted have reported positive benefits similar to those seen in younger patients [12, 19, 20].

While exercise training by HD patients is beneficial, barriers persist to optimizing engagement. Exercise programs remain rare. When they are implemented, they neglect home and community-based activities [11, 13], and the importance of the involvement of family caregivers to support and reinforce exercise [21]. Participation rates are variable, and follow-up exercise adherence and maintenance of improved health outcomes is poor. Thus, exercise has yet to be effectively and systematically incorporated into routine care, particularly amongst older patients [7, 22], leaving renal care misaligned with best practice. Qualitative research exploring this misalignment has identified the need for a shift in the culture of ESRD treatment towards a wellness perspective that includes expectations of exercise participation by older patients during and outside of dialysis treatment, and encouragement by health care practitioners and family caregivers [22]. Creating a HD treatment culture that makes exercise a priority [7] requires that exercise prescription, counseling, and assessment be understood as a part of routine patient care. This signals that exercise, like medications, nutritional monitoring, and fluid management is an expected part of treatment [7, 22, 23]. Staff-level barriers, which have been well identified [16, 22], must be overcome through education to provide training in exercise prescription, lessen fear of potential adverse events, and balance the prioritizing of medical treatment with wellness. Staff must also be educated on how best to provide verbal and non-verbal encouragement to exercise in order to allay patient concerns that staff perceive intradialytic exercise as onerous [23]. Since information about exercise is also a priority for family caregivers in order to alleviate caregiver distress [24], as well as to improve patient health outcomes [25], the neglect of family caregivers in exercise interventions [13, 25] must be redressed.

Educational interventions that facilitate critical self-reflection by health care practitioners, patients, and family caregivers about how contextual factors influence and shape understandings, assumptions, and practices are most effective in changing practice. Yet most knowledge translation strategies fail to effect critical reflection [26, 27]. In response, researchers are turning to the arts, an alternative mode of research dissemination, which fosters critical thinking and reflection, raises social consciousness, and effects change [28–31]. Drama-based methodologies hold particular promise to effect cultures of best practice, but have yet to be utilized to increase exercise in older HD patients. There is increasing empirical support for the effectiveness of research-based drama for learning about health, illness, and patient care in various clinical areas [29, 31–33]. The accessibility and familiarity of film in our contemporary media-driven culture makes it a particularly useful vehicle for engagement of a broad range of professional and lay stakeholders, including patients and family members on complex social issues [34] of which health conditions play a large part.

We describe the protocol for a study we developed to increase exercise participation by older HD patients that involves a drama-based educational intervention. *Fit for Dialysis* is a film based on focus group research with patients, family caregivers, and dialysis staff that identified barriers and facilitators regarding exercise participation and counseling [22]. The film is intended to help effect a shift in the treatment of ESRD towards the inclusion of exercise participation by older HD patients, and exercise encouragement by staff and family caregivers as an expectation of treatment. We will compare the film in addition to a 16-week exercise program implemented in one hospital, with a 16-week exercise-only program in another hospital.

Our study of *Fit for Dialysis* will generate a rich data set for understanding the nature and extent of the impact of the film, for whom it is effective (patients, family caregivers, health care practitioners) and why, and under what conditions. Our study will further facilitate understanding of the actual degree of adoption of exercise, the extent to which and the reasons why the adoption occurred as intended, and the factors important for replication [35]. Further, by using a two-site design, we will generate data that will inform the design of a Phase III, cluster-randomized controlled trial of this intervention.

### Study objectives

The primary objectives are: 1) to qualitatively explore the impact of *Fit for Dialysis* on the knowledge/attitudes of patients, family caregivers, and health care practitioners regarding the importance of exercise-based principles of wellness, and in what ways this is effective in educating, motivating, or sustaining patient exercise during dialysis, at home, and in the community; 2) to quantitatively examine levels of adherence to exercise prescription, and qualitatively explore the factors influencing adherence (e.g. *Fit for Dialysis*, experience of adverse events); and 3) to quantitatively explore the impact of *Fit for Dialysis* on patient physical fitness and activity outcomes.

The secondary objectives are: 1) to qualitatively explore factors related to family caregivers that may impact the successful uptake of the key messages of *Fit for Dialysis*; 2) to qualitatively explore the factors related to nephrologists, nurses, and hospital administrators that may impact the successful uptake of the key messages of *Fit for Dialysis*; and 3) to qualitatively explore factors related to patients’ home and community environments that may impact the successful uptake of the key messages of *Fit for Dialysis*.

## Methods/Design

### The intervention

One hospital will receive the intervention comprised of the *Fit for Dialysis* film and a 16-week exercise program involving activities during dialysis, at home, and in the community (hereafter referred to as the film + exercise hospital). The other hospital will receive the 16-week exercise-only program (hereafter referred to as the exercise-only hospital). For both hospitals there will be a 12-week post-intervention follow-up (i.e. 28 weeks after baseline).

### Fit for dialysis – the film

*Fit for Dialysis* is a 15-min filmed research-based drama that reinforces the importance of exercise counseling and support by nephrology staff and family caregivers, and exercise participation by patients. The film also captures the contextual factors that inhibit exercise during HD, and highlights some of the life circumstances that may influence patients’ self-management of exercise behaviours at home and in the community. It is designed to facilitate critical reflection among health care practitioners about how contextual factors influence and shape direct care practices. Such reflection is intended to assist staff to see how their own practice styles signal underlying assumptions regarding the importance of exercise for wellness vis-à-vis point of care priorities and decisions, and judgments regarding patient safety.

Following patient physical assessments and baseline data collection in the film + exercise hospital, each participant (patient, family caregiver, nephrologist, nurse, administrator, and the physiotherapy assistant (PTA) who will monitor the patients in that hospital) will view *Fit for Dialysis* once; film viewings will be organized by participant group (e.g. patients; nephrologists). To prevent contamination, the physiotherapist (PT) who will conduct assessments and individually tailor the exercise program for patients in both the film + exercise hospital and exercise-only hospital, and the PTA and the research assistant (RA) in the exercise-only hospital, will not view the film.

### Individualized exercise program

The 16-week exercise program is based on a pilot exercise program [19] developed by research team members for the >65 years HD population. It focuses on muscular strength and flexibility, cardiovascular fitness, and functional capacity. The program consists of 20–30 min of cardiovascular exercise 2–3 times per week (40–90 min per week) and 10–15 min of strengthening 2–3 times per week (20–45 min per week) with an intensity of self-reported Rating of Perceived Exertion (RPE) of ‘light’ to ‘somewhat hard’ [36] and the ability to talk without difficulty while exercising. It is based on: (a) an individualized exercise prescription appropriate to each patient’s unique functional status and current activity level (identified by standard PT assessment [37] adjusting for individual participants’ comorbidities; (b) an exercise component, using Thera-Bands™ (rubber resistance tubing) and leg cycle ergometers, to be performed during dialysis sessions and supervised and logged by a PTA; c) an exercise component, using Thera-Bands, and to the greatest extent possible patients’ daily activities and available community resources, to be performed and logged by the patient at home/in the community; and (d) plain language information sheets that identify for patients and family caregivers clear parameters for patient exercise safety.

A registered, experienced PT not affiliated with either hospital and blinded to film versus non-film hospital assignment, will comprehensively assess patients prior to prescribing exercise. The PT assessments will be performed on a 1:1 basis with each patient and will follow a standardized PT core assessment for renal care [38]. The assessments are adapted from the principles of a cardiorespiratory PT assessment [39] and will include: (a) a thorough review of the patient’s medical record, including consultation with attending interprofessional staff (nephrologist, renal nurse, dietician) as needed; and (b) a 45-min physical assessment (including screening tests of the musculoskeletal, neurological, integumentary, cardiovascular, and respiratory systems described below). This approach is essential to ensure the appropriate tailoring of exercise prescription to patient ability, which has been demonstrated to be a successful model within the context of cardiac rehabilitation [40]. Following patient assessments, the PT will also review patients’ physical fitness and activity outcomes and will integrate these data into a tailored exercise prescription for each individual patient. Subsequent changes to patients’ exercise programs (e.g. increasing intensity and duration of exercise) will be made by the PT as needed using the FITT principle (Frequency, Intensity, Time, Type) [41].

Two PTAs not affiliated with either hospital (one for each hospital) will: (a) collect fitness and activity outcome measures for exercise tailoring by the PT and subsequent evaluation; (b) supervise/monitor patients during intradialytic exercise; and (c) document or review activities in the intradialytic and home/community exercise logs. To examine exercise safety, the PTAs will routinely ask patients and staff about the occurrence of any minor non-adverse, and moderate or major adverse events, defined based on categories proposed by Carnes et al [42]. Minor non-adverse events will be defined as exercise-related symptoms that do not necessitate cessation of exercise (e.g. minor and short-term aches and pains). Moderate or major adverse events are defined as those that necessitate a cessation in exercise (e.g. medium to long-term serious event such as angina). Minor non-adverse events will be tracked in the intradialytic and home/community exercise logs which will be reviewed by the PTAs with patients prior to each intradialytic exercise session. For any moderate or major adverse event, the PTAs will defer management to the nurse manager and nephrologist and will consult with the PT to discuss potential exercise modifications.

### Study design

A prospective 2-site parallel intervention trial using mixed qualitative and quantitative methods has been selected to facilitate in-depth exploration of whether and why *Fit For Dialysis* works (or fails) in a particular setting, and for whom, including the actual degree of adoption and the extent to which the adoption occurred as intended.

### Study setting

Hospital A is a dialysis unit serving 318 patients in an acute care hospital in urban central Canada. Hospital B is a dialysis unit serving 210 patients in an acute care hospital in urban central Canada. The two study settings have similar populations of patients and nurse staffing mix. Neither has an existing exercise program as part of their renal program. Both hospitals are fully supportive of this proposal. Both hospitals will receive the exercise program, and an independent researcher will select which of the two sites will receive *Fit for Dialysis* using the following random allocation rule: roll a single 6-sided die and allocate the active intervention site to Hospital A if the number on top of the die is even or to Hospital B if the number is odd [43].

### Participants and inclusion criteria

The recruitment of those deemed eligible to participate will take place over a 12-week period. In-service information sessions for staff will be conducted in the film + exercise and exercise-only hospitals. Invitations to all eligible participants to meet with the RAs to discuss the study will be extended by the nurse manager of each hospital who will also consult with nephrologists to identify eligible patients. Those patients who agree to meet with the RAs will be provided an information letter that describes the study. Informed consent will then be obtained by the RAs from those who have agreed to participate.

The target will be to recruit a total of 60 HD patients (*n* = 30 in the film + exercise hospital, *n* = 30 in the exercise-only hospital). Patients are eligible to participate if they: (a) have conversational ability in English; (b) have a medical diagnosis of HD-dependent ESRD; (c) are a registered HD patient in the film + exercise or exercise-only hospital for at least 3 months; (d) receive ≥ 2 in-centre HD sessions per week; (e) are ≥ 65 years of age; (f) are ambulatory (with or without gait aids); (g) are not currently participating in regular physical activity (structured exercise that includes a cardiovascular and/or strengthening component ≥ 3 times a week for ≥ 10 min per session); (h) are deemed medically eligible by their nephrologist to participate in an exercise program that includes stretching, strengthening, and cardiovascular components; and (i) have a family caregiver who agrees to participate in the study. The film + exercise hospital will view *Fit for Dialysis*. The film + exercise and exercise-only hospitals will participate in the exercise program and keep a log of exercises performed on non-dialysis days. A subgroup of patients (*n* = 20; 10 per hospital) will also participate in semi-structured interviews. Subgroup recruitment will be based on: (1) representation by sex as consistent with the Canadian profile of ESRD patients undergoing HD, which is approximately 60% male and 40% female [44]; and (2) the agreement of a family caregiver to participate in the family caregiver subgroup.

For each participating patient at both hospitals, a family caregiver will be identified. Eligibility criteria include: (a) conversational ability in English; (b) provision of supervision/assistance with activities of daily living and/or emotional support without financial compensation; and (c) related by blood, marriage or adoption to a patient enrolled in the study. Family caregivers in the film + exercise hospital (*n* = 30) will be shown *Fit for Dialysis* and will be given information on the benefits and contraindications of patient exercise, and the safe parameters of patient exercise. Those in the exercise-only hospital (*n* = 30) will only read the information. A subgroup of family caregivers (*n* = 20; 10 per site) will participate in semi-structured interviews. Recruitment will be based on the participation of their relative in the patient subgroup.

We will recruit nephrologists (*n* = 10; 5 at each site) and nurses (*n* = 20; 10 at each site) for semi-structured interviews. Selection will be based on the least and most years of experience within the given discipline. The PTA in the film + exercise hospital site will view *Fit for Dialysis* and will participate in semi-structured interviews (See Table 1 for sources of data collection). The PTA in the exercise-only hospital will participate in semi-structured interviews only. Administrators (*n* = 6; 3 per site) involved in patient management, staff management, and program planning will be eligible to participate in semi-structured interviews. Administrators in the intervention site will also view *Fit for Dialysis*.

**Table 1:** Summary of quantitative and qualitative data sources at specified time points

Quantitative	T_0_: (Baseline)	T_1_:(8 weeks)	T_2_: 16 weeks (End Int.)	T_3_: 12 weeks (Post Int.)
Physical Activity Assessments				
Duke Activity Status Index	●	●	●	●
Pedometer	●	●	●	●
Godin Leisure-Time Exercise Questionnaire^a^	--------------	--------------	--------------	●
Physical Fitness Measures				
Timed Up-and-Go	●	●	●	●
Two-Minute Walk Test	●	●	●	●
Grip Strength Test	●	●	●	●
Exercise Logs^b^				
Intradialytic Log	--------------	--------------	--------------	
Home/Community Log	--------------	--------------	--------------	
Qualitative	T_0_: (Baseline)	T_1_: (8 weeks)	T_2_:16 weeks (End Int.)	
Semi-structured Interviews				
Patients	●	●	●	
Family Caregivers	●	●	●	
Nurses	●	●	●	
Nephrologists	●	●	●	
Administrators	●	●	●	
Physiotherapy Assistants		●	●	

^a^To be completed each week of the intervention, and at 12 weeks post-intervention

^b^To be completed each week of the intervention

### Data collection

Qualitative data collection will include semi-structured interviews. Interviews with participants in both hospitals (patients, family caregivers, nurses, nephrologists, and administrators) at baseline will explore: (1) perceptions/experiences regarding exercise; (2) exercise behaviour; and (3) the barriers and facilitators regarding patient exercise at the level of the individual, unit, organization, and home and community. At 8 and 16 weeks, interviews with participants in both hospitals, and the PTAs will explore: (1) changes in knowledge/attitudes and practice regarding exercise; (2) barriers and facilitators regarding exercise participation and motivating/sustaining patient exercise; and (3) factors influencing adherence to exercise prescription. At 8 and 16 weeks, interviews with the PTA and participants in the film + exercise hospital will additionally explore: (1) perceptions of the effectiveness of *Fit for Dialysis* as an educational modality for educating about, motivating, or sustaining patient exercise frequency or support during dialysis, at home, and in the community; and (2) an in-depth exploration of the role that *Fit for Dialysis* plays in influencing patient exercise adherence.

Quantitative data collection will include the following measures:

- *Demographic Questionnaire* and *Comorbidity Data Form*: Baseline demographic data (e.g. age, gender) will be collected by the RAs from health care practitioners, patients, and family caregivers. Patients’ comorbid illnesses (modified Charlson score [45]) and dialysis history (e.g. etiology of the kidney disease; date that renal replacement therapy was initiated and modality of initial renal replacement therapy; date of initiation of HD if different than date of initiation of renal replacement therapy; history of kidney transplantation, if applicable) will be obtained at baseline by the RAs from patients’ hospital health records, and will be reported to the PT prior to patient assessments.
- *The Two-Minute Walk Test (2MWT)*: The 2MWT measures speed and distance, providing information about functional exercise capacity. Participants are asked to walk as far as they can in 2 min. The assessor uses a digital stopwatch and a calibrated wheel with a counter to measure the distance walked in metres. Two practice walks will be performed before the actual walk test is recorded [46]. The 2MWT has been shown to be reliable and valid in frail older adults, and has been used with the HD population [19, 47]. The 2MWT is our primary physical fitness outcome measure, and will be administered at baseline, 8 and 16 weeks, and 12 weeks after the end of the intervention.
- *The Duke Activity Status Index (DASI)*: The DASI is a simple, easily administered 12-item questionnaire that provides a patient’s self-assessment of functional capacities and can be used to estimate peak metabolic equivalents (a widely utilized measure of exercise intensity). Functional capacity is gauged by the patient’s ability to perform 12 common sets of activities of daily living such as ambulation, housework, and recreation. Responses are weighted; the possible range is from 11 (unable to walk indoors) to 33 (able to do vigorous exercise or aerobics). The DASI has been validated for use with frail and sedentary patients [48, 49], making it particularly useful for the older dialysis population. The DASI will be adminstered at baseline, 8 and 16 weeks, and 12 weeks after the end of the intervention.
- *Grip Strength Test*: The Grip Strength Test is a reliable and valid measure of upper extremity strength and is predictive of mortality and deteriorating health in older adults [50, 51]. Age-specific normative data have been published for grip strength [52]. Grip strength will be measured at baseline, 8 and 16 weeks, and 12 weeks after the end of the intervention in a standardized fashion three times in each arm (alternating between arms) using a Jamar Dynamometer [51]. Since vascular access (e.g. a fistula on the forearm) may interfere with strength testing, the location and type of vascular access will be noted each time the test is administered [53].
- *The Timed-Up-and-Go (TUG)*: The TUG is a test of basic mobility and reflects the ability to transfer from sitting to standing and to walk a short distance (3 m) and return to a seated position [54]. After a practice trial, the final two trials will be recorded and the best measure will be used for analysis [54]. Reliability and validity [55, 56] are well established. The TUG will be administered at baseline, 8 and 16 weeks, and 12 week after the end of the intervention.
- *Godin Leisure-Time Exercise Questionnaire (GLTEQ)*: The GLTEQ is a simple 3-item measure assessing the frequency of mild, moderate, and strenuous bouts of exercise performed for at least 15 min in duration in a typical week. It will be used to capture a range of activities undertaken by patients (e.g. housework, recreational activities), as well as to compensate for the inability of the pedometer to capture exercise and activity undertaken in reclined positions during HD treatment, and possible non-adherence with wearing the pedometer. Co-I SA has successfully used this measure in exercise trials with adult cancer patients [57, 58]. It has also been validated in studies with older adults with other chronic illnesses [59–61]. On a weekly basis throughout the 16-week intervention, and at 12 weeks after the end of the intervention, the PTAs will contact patients by phone to record how much exercise was done that week using the GLTEQ [62].
- *Pedometer*: Pedometers are a widely used method for inferring activity levels based on the number of steps taken throughout the day [63, 64] and considered the gold standard in physical activity measurement [65]. Patients will be asked to wear a pedometer for 1 week at baseline, and at the 8- and 16-week assessments, and 12 weeks after the end of the intervention (to facilitate comparison with the GLTEQ measured at those time points). The PTAs will download the data from the pedometer at each assessment point. Compliance with wearing a pedometer, and its validity as a measure of exercise have both been demonstrated in older adults with various chronic illnesses [64, 66].
- *Exercise Logs*: During the 16-week intervention, an intradialytic exercise log will be kept by the PTAs documenting exercises performed by the patients, as well as perceived exertion and heart rate. Patients will also be asked to keep a log of the exercises performed at home and in the community. The PTAs will review the exercise logs with the patients prior to each intradialytic exercise session to discuss any concerns.
- *Adherence*: Adherence is important to assess as it is a necessary step to achieve gains in physical fitness (which would, in turn, lead to broader health benefits). However, there is no gold standard for assessing adherence to exercise, particularly in an elderly dialysis population. We have based our exercise prescription and definition of adherence on clinical judgement and prior experience providing exercise to frail older HD patients [19]. Adherence is thus defined as meeting at least 70% of the exercise prescription (recognizing that the number of minutes of prescribed exercise per week may change during the protocol as patients improve their fitness level with exercise), on average, over the 16-week intervention [67]. Given the separate components of exercise (intradialytic and home/community-based) that are being targeted by our intervention, we will include both a global adherence measure and separate adherence measures for the two exercise components (intradialytic and home/community). This same definition of adherence will be used for the intradialytic exercise and home/community exercise components. In addition, a global dichotomous measure of adherence will be used to facilitate inclusion in a multivariable model in which patients will be considered adherent overall if they are adherent (at least 70% of the exercise prescription) to both intradialytic and home/community exercise components, and non-adherent otherwise.

### Sample size

Sixty patients (30 in the film + exercise hospital, 30 in the exercise-only hospital) will be recruited. Thirty patients per study site is the minimum recommended for early phase studies to provide reasonable estimates of efficacy on outcomes (estimating means and variances) [68–70]. A total sample size of 60 will facilitate building a multivariable model with up to 3 covariates plus 3 main factors (hospital, time, and hospital X time). Moreover, with alpha = 0.05 and power of 80%, we would need about 30 patients per arm to detect a 0.65 SD difference between hospitals in the 2MWT, our primary quantitative outcome. A 0.65 SD difference represents a moderate effect size and is equivalent to about 22 m, which is larger than the minimum clinically important difference for the 2MWT [71]. Based on the pilot study with older HD patients [19] conducted by several co-investigators, there was 0 attrition over the duration of the exercise program. Although we recognize that the observed attrition rate may be higher than this in the present study, estimating attrition is a specific aspect of feasibility that we will be evaluating in this phase of the research. Sample size calculations for early phase studies commonly do not include adjustments for attrition [68–70].

Patients (*n* = 20; 10 per site), family caregivers (*n* = 20; 10 per site), nephrologists (*n* = 10; 5 at each site) and nurses (*n* = 20; 10 at each site) will be selected to participate in qualitative semi-structured interviews (Section 4.5.1). Sample sizes are consistent with what is deemed sufficient for qualitative sampling in a homogeneous group within a single setting, and provides a sufficient number of participants to facilitate saturation [72].

### Analysis

Qualitative analysis will begin with an inductive descriptive process of sorting and defining the data [73]. Descriptive codes of analysis are attached to text segments, which will then be grouped into broad topic-oriented categories related to knowledge/attitudes, preferences and practices related to exercise, and barriers and facilitators regarding exercise participation and support. Text segments belonging to the same category will be compared within and across all participant interviews both within and between hospitals. Topic-oriented categories will be further refined and formulated into fewer analytical categories through an inductive, iterative process. The research team will develop the initial coding scheme. Each will independently open-code 25% [74] of interview transcripts, and then will meet to compare applied codes and to ensure consistency in interpretation. The remaining data will then be coded using NVivo 11 [75], a qualitative analysis software program. An audit trail will ensure analytic reliability through detailing: theoretical assumptions; codes, concepts and models; methodological and analytic procedures; and reflections on data collection. The audit trail will be reviewed by the research team to ensure analyses are supported by data [76]. In addition, two strategies to ensure internal and external validity will be used: triangulation [73]; and rich, thick description [77]. First, data triangulation involves using different data sets (e.g. family caregiver and patient interviews) to build coherent justification of interpretation. For example, if a patient discloses during the baseline interview that she is sedentary at home because of the injury-related fears of her family caregiver, but at 8 weeks demonstrates a significant improvement in community-based exercise, the patient’s and family caregiver’s interview transcripts will be analyzed to identify which specific components of *Fit for Dialysis* proved effective for the family caregiver at overcoming deficiencies in knowledge of the safety of exercise in settings beyond the outpatient clinic. Second, rich, thick description will be facilitated through the provision of verbatim quotes to allow readers to assess the strength of the relation between data and analysis.

Quantitative analysis of exercise prescription adherence (intradialytic and home/community exercise components) will be examined. To determine if patients are adherent to the intradialytic component, PTA-recorded minutes of intradialytic exercise performed each week will be compared to the prescription for that week (e.g. 50 min in week 3 were done, 60 min were prescribed, which is 83% adherent). Similarly, for the home/community component, adherence will be based on weekly number of minutes of exercise performed based on the GLTEQ results compared to the weekly exercise prescription. Multivariable logistic regression for each adherence variable will be performed at 8 weeks (mid-intervention), 16 weeks (end of intervention), and 12 weeks post-intervention including hospital as the main predictor. Covariates including age and sex will be included in the model based on clinical judgment guided by our prior work [19] and the literature.

To explore the impact of *Fit for Dialysis* on patient fitness and activity outcomes, while keeping in mind the phase of research, our focus is to estimate change in each of the 6 measures using a hospital X time linear repeated measures model for each outcome. The 2MWT is our primary outcome. Examining hospital-level effects will allow us to determine if the two hospitals were similar at baseline. Examining time effects within each hospital will provide information on the effects of the intervention in that hospital (film + exercise or exercise-only). Additionally, examining the hospital X time interaction will allow us to determine the additional effect of the film in addition to the exercise intervention. Since this is an early-phase intervention trial and it is unclear which of our quantitative outcomes will be most responsive in this population, we will also examine the responsiveness of each of the 6 outcome measures using standardized response mean to gauge whether the film + exercise is associated with a small, moderate, or large effect size [78] for each outcome. Covariates will be similar to Primary Objective 2. To examine the impact of adherence on the 2MWT, we will include a dichotomous variable reflecting global (intradialytic and home/community-based exercise considered together) adherence (yes/no). We will also explore including the two separate adherence components (intradialytic and home/community) in the repeated measures model, recognizing the risks of collinearity and model over-fitting (with 8 variables and 60 patients, including an interaction term between the two adherence variables).

As an exploratory analysis to get an initial sense of the temporal effects of both the film and the exercise program, we will calculate change scores for each of the 6 measures from baseline to 8 weeks, from 8 to 16 weeks, and from 16 weeks to 12 weeks after the end of the intervention (i.e. 28 weeks after baseline). An additional exploratory analysis will examine the six outcome measures (DASI, GLTEQ, TUG, 2MWT, Grip Strength, and pedometer) at 12 weeks after the end of the intervention. This analysis will consist of a linear repeated measures model similar to above, with two time points (8 and 16 weeks) and 12 weeks follow-up, and will examine whether any observed gains during the intervention period persist, continue to improve further, or become attenuated over time in both the film + exercise and exercise-only hospitals.

## Discussion

ESRD in older Canadians is a growing health concern with significant quality of life implications. While exercise can significantly improve many of the negative sequelae and poor health outcomes associated with ESRD [8, 13], it has yet to be effectively and systematically implemented into dialysis care [7, 22]. Our intervention stands to markedly improve care for older persons with ESRD by effectively bridging research on exercise with dialysis care. Our exploration of the impact of *Fit for Dialysis*, for whom it is effective (patients, family caregivers, health care practitioners), why, and under what conditions, is poised to make an important contribution to the development and evaluation of a theoretical base to support the choice and development of film as an educational intervention for health care providers, patients, and family caregivers. This will inform the development of a scientific basis to support the implementation of best evidence in HD practice and, more broadly, other arts-based knowledge translation initiatives. The study will further generate a rich data set for understanding the actual degree of adoption of exercise, the extent to which and the reasons why the adoption occurred as intended, and the factors important for replication [35]. Findings from this study are intended to inform the future development of a Phase III cluster-randomized trial of the intervention. If *Fit for Dialysis* is proven beneficial to patients, nephrology staff and family caregivers, the model could be used to support the National Kidney Foundation’s guideline recommendation (NKF-KDOQI) [79] that exercise be incorporated into the care and treatment of dialysis patients.

## Abbreviations

2MWT: Two-Minute Walk Test
DASI: Duke Activity Status Index
ESRD: End-stage renal disease
GLTEQ: Godin Leisure-Time Exercise Questionnaire
HD: Hemodialysis
PT: Physiotherapist
PTA: Physiotherapy assistant
RA: Research assistant
RPE: Rating of Perceived Exertion
TUG: Timed-Up-and-Go
